# Differentiating Neuroblastoma: A Systematic Review of the Retinoic Acid, Its Derivatives, and Synergistic Interactions

**DOI:** 10.3390/jpm11030211

**Published:** 2021-03-16

**Authors:** Nadiya Bayeva, Erin Coll, Olga Piskareva

**Affiliations:** 1 Cancer Bio-Engineering Group, Department of Anatomy and Regenerative Medicine, RCSI University of Medicine and Health Sciences, D02 YN77 Dublin, Ireland; nadiyabayeva@rcsi.ie (N.B.); erincoll20@rcsi.ie (E.C.); 2 School of Pharmacy and Biomolecular Sciences, RCSI University of Medicine and Health Sciences, D02 YN77 Dublin, Ireland; 3 National Children’s Research Centre, Our Lady’s Children’s Hospital Crumlin, D12 8MGH Dublin, Ireland

**Keywords:** neuroblastoma, retinoic acid, combinational therapy

## Abstract

A neuroblastoma (NB) is a solid paediatric tumour arising from undifferentiated neuronal cells. Despite the recent advances in disease management and treatment, it remains one of the leading causes of childhood cancer deaths, thereby necessitating the development of new therapeutic agents and regimens. Retinoic acid (RA), a vitamin A derivative, is a promising agent that can induce differentiation in NB cells. Its isoform, 13-cis RA or isotretinoin, is used in NB therapy; however, its effectiveness is limited to treating a minimal residual disease as maintenance therapy. As such, research focuses on RA derivatives that might increase the anti-NB action or explores the potential synergy between RA and other classes of drugs, such as cellular processes mediators, epigenetic modifiers, and immune modulators. This review summarises the in vitro, in vivo, and clinical data of RA, its derivatives, and synergising compounds, thereby establishing the most promising RA derivatives and combinations of RA for further investigation.

## 1. Introduction

A neuroblastoma (NB) is an aggressive heterogeneous solid tumour arising from the sympathetic nervous system’s precursor or immature cells during embryonic development or early postnatal life [1,2]. Neuroblastoma accounts for approximately 8% of malignancies in patients younger than 15 years of age, but results in a disparate 15% of paediatric cancer deaths. The disease is often diagnosed at late stages, as 50% of the newly registered cases display metastases in regional lymph nodes, bone marrow, bone, liver, and skin [3,4].

NB is highly heterogenic clinically, and the disease progression and outcome depend on many risk factors, including the age at diagnosis, tumour size and localisation, histopathologic classification (e.g., stroma cell differentiation), genetic abnormalities, and *MYCN* status [1]. Newly diagnosed patients are stratified into “risk groups” based on the combination of the listed risk factors and the likelihood of developing an aggressive form of neuroblastoma. The standard treatment plan consists of chemotherapy, surgical resection, and/or radiotherapy [5,6]. The patients receive a means of maintenance therapy to eradicate minimal disease and prevent NB recurrence, such as isotretinoin, dinutuximab, GM-CSF, or IL-2. However, despite recent treatment advances, the patients’ five-year survival rate in the high-risk group and with refractory neuroblastoma remains 40–50% [7]. 

Cell fate determination and differentiation are crucial in assigning cells to their functional roles in any given tissue. Differentiation, however, is largely impaired in neuroblastoma, wherein immature cells of the sympathetic nervous system, known as the neural crest cells, lose the ability to differentiate and fail to develop into mature cells at some point. These neural crest cells give rise to stromal Schwann and neuroblastic cells that are present at varying degrees of differentiation in neuroblastoma tissue. Schwann cells’ differentiation degree and the tumour grade serve as predictive biomarkers for disease outcome by classifying tumour into three subtypes—undifferentiated, poorly differentiated, and differentiating [8]. Furthermore, the loss of Schwann cell precursor differentiation into bridge cells is a hallmark of high-risk NB [9].

Many processes rely on the retinoic acid signalling pathway through heterodimerisation of the retinoic acid receptor (RAR) and retinoid X receptor (RXR) groups. The retinoic acid (RA) signalling pathway plays a vital role in early embryo development and neurodevelopment. In this context, neuroblastoma, which derives from immature nerve cells, is often seen as a disease with a broken RA pathway. Repairing differentiation in neuroblastoma could potentially halt disease progression and improve the patient outlook. 

Isotretinoin, also known as 13-cis-retinoic acid, is currently used as part of the treatment regimen for high-risk neuroblastoma patients and induces some neuroblastoma cell lines to differentiate [10,11,12]. As an essential metabolite of vitamin A, it plays a critical role in cellular proliferation, differentiation, and organogenesis [13]. The use of RA as maintenance or combinational therapy aims to reduce the Schwann cell precursor’s ability to lead to recurrent or drug-resistant tumours. 

This review summarises RA’s current data, its metabolites, its derivatives, and drugs that modulate its actions published from 1 January 1980 to 1 July 2020.

## 2. Materials and Methods

The articles investigated in this review were obtained by searched PubMed databases using the keywords “retinoic acid” and “neuroblastoma”. The search was limited to original papers published in the English language, clinical trials, comparative studies, observational studies, system reviews, and meta-reviews published between 1 January 1980 and 1 July 2020. The initial search yielded 331 articles. After screening titles and abstracts, 87 relevant primary studies were identified and included in the review. An additional 15 studies were recovered after analysing the review articles on retinoic acid derivatives. The information about using retinoic acid and its derivatives in clinical trials was obtained from PubMed and ClinicalTrials.gov databases. The initial search in PubMed databases identified 12 randomised control trials of interest, all of which were reviewed in this article. The currently undergoing studies on the retinoic acid were accessed in ClinicalTrials.gov databases, resulting in 41 clinical trials that either supported the previously identified PubMed published studies were actively recruiting, terminated, active, or compete without yielding statistical data. 

### 2.1. Data Sources and Search Strategy

We carried out a systematic review adopting the Preferred Reporting Items for Systematic Reviews and Meta-Analyses (PRISMA) guidelines [14], while the search steps were performed according to the Cochrane handbook for systematic reviews [14]. The relevant original studies were identified through PubMed database searches; the current trials concerning RA treatment for neuroblastoma were extracted from clinicalgov.org database. We used the following MeSH Major Topic key words in the search strategy: “neuroblastoma”, “retinoic acid”, “vitamin A”, “isotretinoin”, and “tretinoin”. The last search was performed on 1 July 2020. The identified articles were then screened for additional relevant articles.

### 2.2. Study Selection

Two authors (N.B. and E.C.) accessed the titles and abstracts of the retrieved publications for inclusion into the study, with the assistance of the second author (O.P.) in case of discrepancies. 

### 2.3. Inclusion and Exclusion Criteria

The studies selected for this review followed these inclusion criteria: (1) randomized control trials, clinical trials, comparative studies, and primary research papers; (2) peer-reviewed studies published in English with full text available; (3) studies using the human/animal cell lines.

The exclusion criteria were as follows: (1) reviews and meta-analysis papers, case reports, letters to the editor, and conference/meetings abstracts; (2) non-English publications; (3) non-full text articles; (4) using exclusively biological techniques/treatment (including silencing of genes via mRNA/ssRNA/CrisprCas9 and use of antibodies); (5) examining the role of RA signalling in organism development.

### 2.4. Data Extraction

The author (N.B.) extracted data from the papers included and entered them into an Excel worksheet. Any disagreement was resolved through discussion between the authors (N.B., E.C., and O.P). The following information were recorded in the Excel form: data about the publication (year of publication, author’s name, journal), investigated treatment (name, dose, route of entry), experimental model (in vitro/in vivo, cell/animal line, size of the population), experimental results (change in cell viability/morphology, change in tumour growth rate, quantitation of maturity, lineage markers, and differentiation features (increased expression of TUBB3, NSE, GAP-43, and NeuN)), and phenotypic changes induced by differentiation markers (e.g., neurite outgrowth).

## 3. Results

### 3.1. Search Results

**Figure 1:**
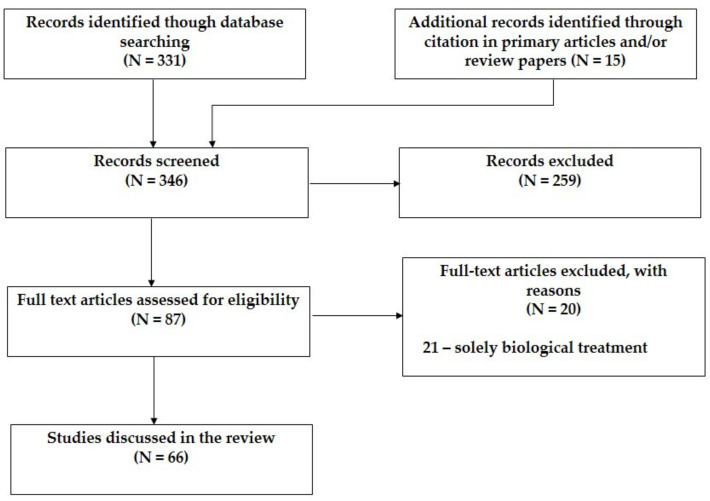
PRISMA flow diagram of the study selection process.

Three-hundred and thirty-one articles were yielded initially through an online database search, while an additional 15 papers were identified through examining the references of the review papers. The resulting 346 articles were then evaluated based on title, abstract, and keywords, which led to the exclusion of 259 articles. The full text of the selected 87 articles was accessed, which resulted in the exclusion of 21 articles that focused solely on biological treatment. Finally, 66 articles were selected and included in the review. Out of those, 54 were primary articles and 12 were randomized clinical trials. Our selection strategy for original research is illustrated with the appropriate flow diagram in Figure 1.

### 3.2. Retinoic Acid for Neuroblastoma Treatment

**Figure 2:**
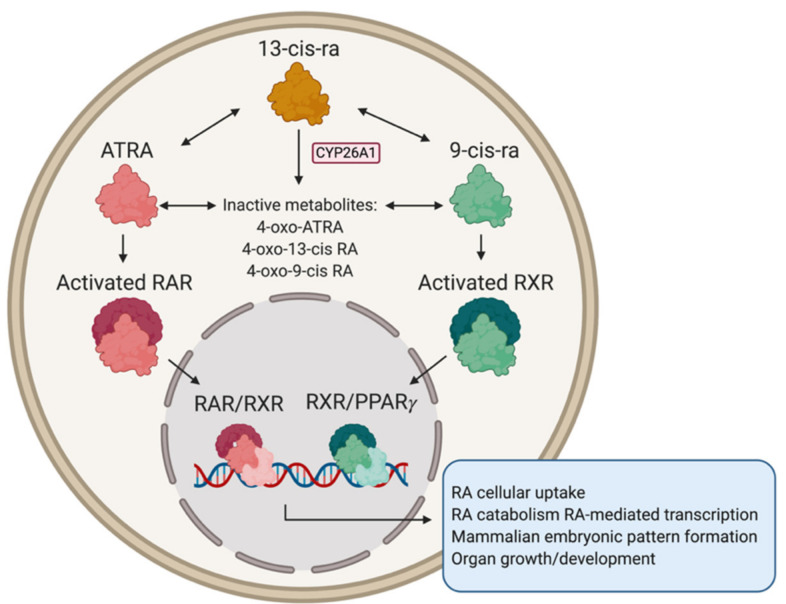
Signalling cascade of RA isoforms. Created with BioRender.com (accessed on 9 February 2021).

Derived from retinol through two oxidation reactions, RA is found in three isomers: all-trans RA (ATRA), 13-cis RA, and 9-cis RA. ATRA, the most predominant form in the cells, is responsible for gene expression change through activation of RAR (Figure 2). Both deficiency and excess of vitamin A can cause foetal malformations and adverse neurodevelopmental outcomes. 

Applied to neuroblastoma cells, ATRA induces neurite formation and outgrowth, which are the hallmarks of neuronal differentiation [15]. 13-cis RA is the less abundant RA isoform. Unlike the other RA isomers, 13-cis RA is weakly associated with RAR and RXR and requires larger concentrations to induce NB cell differentiation; still, it is the form currently preferred for NB treatment due to its favourable pharmacokinetic profile [16,17]. The last commonly described isoform of RA is 9-cis RA. This form, naturally produced only in excess RA conditions, possesses the greatest affinity to RXR. 

Among the listed RA isoforms, 9-cis RA could be considered the most potent inducing NB cell differentiation in vitro. Compared to ATRA, 9-cis RA induces the morphological indicators of neuronal differentiation of *MYCN* amplified (MNA) cells more rapidly [18] and leads to a more significant decrease in cellular proliferation in *MYCN* non-amplified (non-MNA) cells [17]. Furthermore, it accounts for the most significant expression of NB differentiation markers (AChE production, *RAR-a*, and *RAR-b* mRNA) and the decreased expression of MYCN [18]. The observed difference in RA isoform efficiency is attributed to the 9-cis RA activation of RXR, as LG153 and LG53, RXR-selective analogues, can generate the same effects as 9-cis RA, although to the lesser degree [19,20]. However, Ponthan et al. demonstrated that 9-cis RA treated cells were undergoing apoptosis after the treatment course [21]. Further in vivo studies using xenografted mice showed that 9-cis RA, while strongly inhibited the tumour growth, led to a marked weight loss in treated animals [22] (discussed in Section 3.9). The exhibited toxicity of 9-cis RA and its short half-life and the low bioavailability, limit the therapeutic use of this compound [22].

Oxo-metabolites of RA, as products of RA inactivation (Figure 2), are generally thought to lack effect on NB cells, yet 4-oxo-13-cis RA was found to induce NB differentiation and a proliferation arrest similar in effectiveness to 13-cis RA when tested in both MNA (SMS-KCNR, SMS-KCN, SMS-KANR, and SK-N-BE(2)) and non-MNA (CHLA-79, CHLA-20, and SMS-LHN) NB cell lines [23]. This compound induced neurite outgrowth, cell proliferation arrest, and increased expression of *RAR-b, NeuN,* and *Map2c*; and decreased *MYCN* on the transcriptional level at the same magnitude as 13-cis RA. Of note, both 4-oxo-13-cis RA and 13-cis RA elicited a greater response in MNA NB cell lines. 

### 3.3. Retinoic Acid Derivatives for Neuroblastoma Treatment

The impacts of RA on cell proliferation and differentiation prompted an examination of several RA derivatives as candidates to increase the potency of RA signalling. One of the investigated compounds was Ro13-6307, a derivative of 9-cis RA (Table 1). This drug shares the antiproliferative and pro-differentiative properties of the parental compound; it decreased the tumour volume and weight in mice xenografted with SH-SY5Y cells. However, the data on the compound toxicity profile were contradictory. The initial studies revealed that the animals exhibited significant weight loss, a sign of severe drug toxicity, after daily administration of 0.3 mg/animal of Ro13-6307 [22]. Contrarily, in the subsequent experiment, the animals treated with 0.12 mg/animal of Ro13-6307, a dose sufficient to elicit an anti-tumour effect, gained weight slower than those in the control group but exhibited no other signs of toxicity [24]. These results suggest that this compound could be considered a candidate for clinical trials in NB treatments while signifying the importance of toxicological screening and establishing a safe dose due to the low therapeutic index of the investigated drugs. 

The most effective derivative of RA currently used in clinical practice is fenretinide. Unlike RA, it exhibited no impact on NB cell differentiation; instead, fenretinide’s action is mediated by ceramide accumulation and the production of reactive oxygen species, leading to sustained activation of JNK/p38 MAPK pathways and apoptosis [25,26]. Its pro-apoptotic effect was reported in RA-resistant neuroblastoma cell lines LA-N-5, SMS-KCNR, and SMS-LHN [27]. Furthermore, fenretinide and CD437 synergized with commonly used cytotoxic drugs, cisplatin, etoposide, and carboplatin, enhancing the apoptosis of non-MNA SH-SY5Y, MNA SK-N-BE(2), and LAN5 neuroblastoma cell lines [28]. While fenretinide alone is a potent anti-cancer drug, its simultaneous use with RA might have limited efficiency in the clinic. The treatment with 1 μM ATRA 5 days before fenretinide exposure led to decreased number of cellular deaths in NGP, SK-N-BE(2c), and SH-SY5Y cell lines, while enhancing fenretinide pro-apoptotic actions on IMR-32 cell line [29].

In addition to fenretinide, other derivatives of 9-cis RA might be of interest for treating NB. One of them, 9-cis-UAB30, was found to induce neuronal differentiation in both MNA and non-MNA cell lines, with 10 µM of the drug increasing the neurites’ outgrowth by 100% (SH-SY5Y), 200% (SK-N-AS, SK-N-BE(2), SH-EP, and WAC(2)), and 400% (IMR-32) [30]. This compound also exhibited an anti-tumour effect in animal models which was more potent than 13-cis RA. Oral administration of 9-cis-UAB30 to SK-N-AS or SK-N-BE(2) xenografted mice decreased the tumour weight by 50% or 80%, respectively, as compared to 13-cis RA treated animals. The similar pro-differentiating and anti-tumour properties are shared by 9-cis-UAB30 derivative 6-methyl-UAB30 (6-Me). Both MNA WAC(2) and SK-N-BE(2), and non-MNA SH-EP and SK-N-AS cells, treated with 25 μM of 6-Me, demonstrated a 200–225% increase in neurite growth and a 25–75% decrease in expression of stem cell markers *Oct4*, *Nanog*, and *Sox2* [31]. Furthermore, the compound maintained its anti-NB actions *in vivo*, decreasing the tumour volume by 40% and increasing the 30-day survival rate by 100% in SK-N-BE xenografted mice.

Retinoic acid-triazolyl compounds are other RA derivatives exhibiting potent anti-NB effects. Out of the investigated chemicals, (2E,4E,6E,8E)-(1-(2-nitrophenyl)-1H-1,2,3-triazol-4-yl)methyl 3,7-dimethyl-9-(2,6,6-trimethylcyclohex-1-en-1-yl)nona-2,4,6,8-tetraenoate and (2E,4E,6E,8E)-(1-(2-fluorophenyl)-1H-1,2,3-triazol-4-yl)methyl 3,7-dimethyl-9-(2,6,6-trimethylcyclohex-1-en-1-yl)deca-2,4,6,8-tetraenoate inducted differentiation in NB cells more effectively than ATRA, both leading to a 2.6-fold greater increase in the expression of maturation marker NeuN in murine the Neuro2a line compared to non-treated cells [32]. Cyclic retinoic geranylgeranoic acid (10 μM) also caused NB cell differentiation, coinciding with a 50% decrease in cellular proliferation compared with ATRA-treated SH-SY-5Y cells [33]. Another compound, all-trans-retinoyl β-glucuronide (RAG), was found to be efficient in inducing MNA NB cell differentiation. Exposure to 7.5 μM RAG was sufficient to cause neurite outgrowth and proliferation arrest in LAN-5 cells [34]. Moreover, RAG administration decreased the tumour incidence and tumour progression rates in the treatment group composed of 10 LAN5 xenografted mice [34]. The fact that the treated animals experienced no toxicity implicates this compound as a potential candidate for anti-NB treatment [35].

While not being investigated explicitly for neuroblastoma, several retinoic derivatives were found to induce differentiation in other cancer types. Two of those compounds affecting cancer cells are N-(4-hydroxyphenyl) amido (4HPTTNPB) and 4-hydroxybenzyl (4HBTTNPB). They were derived from TTNPB, an RXR receptor agonist that is also an extremely potent teratogen [36]. Both its derivatives exerted pro-apoptotic action on the breast cancer MCF-7 line, where treatment with 10 nM of 4HPTTNPB resulted in a 10–20-fold increase in the induction of pro-apoptotic markers ATF3, GADD34, BBC3, and NOXA, marking its great potential as an anti-cancer treatment [37]. 

**Table 1:** Chemical structures and mechanisms of action of RA metabolites and derivatives tested using neuroblastoma (NB) cell lines.

Name of the Compound	Chemical Structure	Mechanism of Action	Reference
Fenretinide	N-(4-Hydroxyphenyl)retinamide	Low affinity to RAR/RXR, ceramide accumulation	[16,27,28]
9-cis-UAB30	(2E,4E,6Z,8E)-8-(3,4-dihydro-2H-naphthalen-1-ylidene)-3,7-dimethylocta-2,4,6-trienoic acid	RXR agonist	[30]
6-Methyl-UAB30	(2E,4E,6Z,8E)-8-(3,4-dihydro-2H-naphthalen-1-ylidene)-3,6,7-trimethylocta-2,4,6-trienoic acid	RXR agonist	[31]
LG153	4-[1-(3-Chloro-5,5,8,8-tetramethyl-5,6,7,8-tetrahydro-naphthalen-2-yl)-vinyl]-benzoic acid	RXR agonist	[20]
LG69, or Bexaroten	4-[1-(3,5,5,8,8-pentamethyl-6,7-dihydronaphthalen-2-yl)ethenyl]benzoic acid	RXR agonist	[19]
CD437, or AHPN	Naphthoic acid	RAR agonist	[28]
Ro 13-6307	Ethyltetrahydrotetramethyl-2-naphthalenyl-3-methyloctatrienoic acid	RXR agonist	[22,24]
RA-Triazolyl derivatives	RA-Triazolyl Derivatives	Not specified	[32]
Geranylgeranoic acid (GGA)	acyclic retinoid, natural diterpenoid	RXR agonist	[33]
All-trans-retinoyl β-glucuronide	All-trans-retinoyl β-glucuronide	Not specified	[34]
TTNPB (Ro 13-7410)	(E)-4-[2-(5,6,7,8-tetrahydro-5,5,8,8-tetramethyl-2-naphthylenyl)-1 -propenyl] benzoic acid	RAR agonist	[37]
N-(4-hydroxyphenyl)amido (4HPTTNPB)	Hydroxyphenyl modification of TTNB	Low affinity to RAR/RXR	[37]
4-hydroxybenzyl (4HBTTNPB)	Hydroxybenzyl modification of TTNB	Low affinity to RAR/RXR	[37]

### 3.4. Inhibitors of CYP26

In a cell, RA-mediated RAR activation results in increased expression of the CYP26 enzymes. Those enzymes are primarily important for metabolism and inactivation of all RA isoforms, so their activation acts as a negative regulator on the RA pathway. Contrarily, an inhibition of those enzymes could lead to the amplification of RA signalling. Testing this theory, the effect of R116010, an inhibitor of CYP26, on RA-mediated differentiation of NB cells, was investigated [38]. The simultaneous exposure of those cell lines to R116010 compound, and RA (ATRA and 13-cis RA) increased the levels of cellular ATRA concentration compared to ATRA or 13-cis RA treatment alone. In turn, the higher levels of ATRA enhanced the expression of maturating neuronal markers (CRABPII, RARβ) and downregulated MYCN expression in SH-SY-5Y and NGP cells. 

Conversely, SH-EP and GIMEN cell lines, which exhibited the constant levels of CYP26 expression following ATRA treatment, did not benefit from additional R116010 treatment. Interestingly, R116010 alone increased *MYCN* mRNA expression in the MNA NGP cell line, but the mechanism of this change is not understood. Furthermore, the compound was retained its properties in vivo when tested in the immunodeficient athymic nude mice model. In animals bearing SH-SY-5Y xenografts, the administration of R116010 before ATRA and 13-cis RA resulted in increased plasma concentrations of RA (2.3-fold and 4.6-fold increases, respectively) [38].

Inhibition of RA metabolism also contributes to the neuroprotective action of the tetracycline antibiotic minocycline. This drug alone inhibited RA metabolism and enhanced RA-activated β-galactosidase activity in RA-pre-treated human and murine NB cells in vitro [39].

### 3.5. Drugs That Are Derivatives of Other Vitamins

Vitamin A and vitamin D share similar properties as fat-soluble compounds that activate the same nuclear RXR receptor. Therefore, there is a high likelihood of vitamin D derivatives attenuating RA signalling or sharing RA properties. Stio et al. examined the impacts of 1,25-dihydroxyvitamin D3, 24,25(OH)2D3, KH 1060, and EB 1089 on SH-SY-5Y NB cells [40]. Out of four tested compounds, 1,25-dihydroxyvitamin D3 and EB 1089 caused the most significant decreases in cellular proliferation when cotreated with 9-cis RA, resulting in MYCN protein levels being decreased by an additional 15%. 

While vitamin E does not share a signalling pathway with vitamin A, it is still implicated in cancer prevention due to its antioxidative action. Nevertheless, Trolox, a vitamin E analogue, did not affect ATRA-treated non-MNA SH-SY-5Y cells [41]. On the other hand, n-acetyl cysteine, another antioxidant, has decreased cellular proliferation and induced cellular apoptosis when used with ATRA. This effect was attributed to the n-acetyl cysteine-induced inhibitor of glutathione synthesis. A similar decrease of cell proliferation and apoptosis was observed during ATRA exposure in combination with BSO, a specific glutathione synthesis inhibitor. These findings suggest that glutathione synthesis inhibition potentiates RA-mediated cell death and might become an addition to the NB treatment protocol. However, the results of this study might be limited by the absence of traditional NB differentiation markers [41].

### 3.6. Drugs Activating the Cellular Cascades That Potentiate RA Efficiency

**Figure 3:**
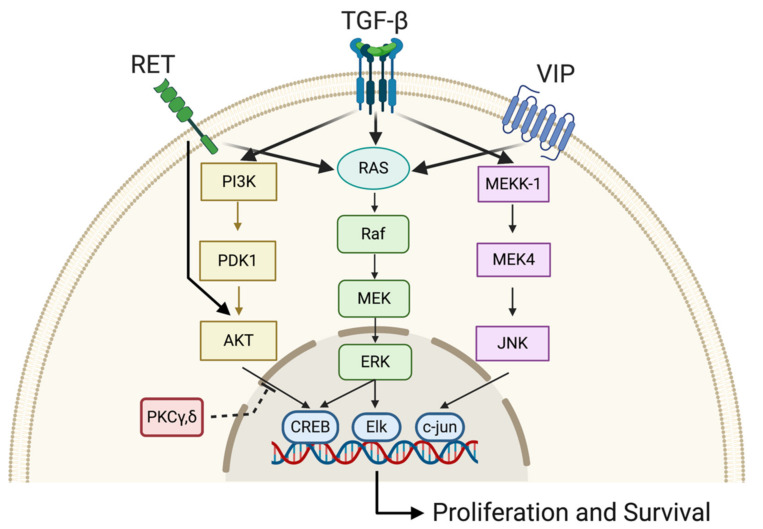
Schematic of the pathways interacting with the RA signalling cascade. Created with BioRender.com (accessed on 9 February 2021).

RA exerts its major cellular effects through activating nuclear receptors and initiating changes in gene expression. However, its downstream signalling elements interact extensively with other signalling pathway cascades (Figure 3). Thus, the compounds that mediate those pathways might affect RA action in neuroblastoma (summarised in Table 2). In some cases, their interaction might lead to significantly increased NB cell susceptibility to RA effects. For example, the cotreatment of ATRA and U0126, a MEK inhibitor, was found to decrease cell proliferation of shNF1 SH-SY5Y cell line, which is otherwise resistant to RA-mediated proliferation arrest [42]. 

On the contrary, in parental SHSY5Y inhibition of MEK results in the diminished effects of ATRA, as was evident in the experiment with PD98059. Administered before ATRA treatment, this compound counteracted RA-mediated neurite formation [43]. In addition to MEK, other mitogen-activated protein kinase (MAPK) pathway members might also influence RA signalling cascade. 

Studies report contradictory effects of c-Jun N-terminal kinase (JNK) signalling inhibition on RA cellular action. SB203580, JNK1/2 inhibitor, exhibited no effect on the RA-treated NB cells, but curcumin, which also interferes with the JNK pathway, blocks RA-mediated NB differentiation without causing cellular proliferation arrest [41,44]. While studies support coordination between RA and MAPK signalling pathways, activation of other pathways, such as phosphatidylinositol 3-kinase (PI3K), might also require the cells to undergo RA-induced NB differentiation. The inhibition of this pathway, achieved via LY294002, abolished RA-induced differentiation markers p-AKT and p21 and morphological differentiation of SK-N-SH and BE(2)-C cells [45]. The activation of MAPK/PI3K pathways is involved in the observed synergistic effects of RA and ciliary neurotrophic factor (CTNF). Exposure of RA-pre-treated SHSY5Y cells to 50 and 500 nM CTNF, while increasing neurite outgrowth and neuronal survival in a dose-dependent manner, also enhanced the expression MAPK and PI3K pathway activity markers P-STAT3, P-Akt, and P-ERK1/2. Furthermore, interference with inhibitors of JAK2, STAT3, and PI3K resulted in the loss of the observed pro-differentiation effects [46].

In addition to PI3K, vascular endothelial growth factor receptor 2 (VEGFR2), epidermal growth factor receptor (EGFR), and rearranged during transfection (RET) pathways were also found to be essential for RA-mediated differentiation. Their antagonist, Vandetanib, impedes neurite formation in RA-treated SH-SY5Y, SKN-BE2n, SKN-BE2c cell lines at concentration 5 μmol/L [47]. While Vandetanib affects a broad range of cellular cascades, the examined interference with RA signalling might be attributed to the effects of RET pathway. A combination of ATRA with D4 aptamer, a construct binding specifically to the RET receptors, inhibited neurite outgrowth and decreased the expression of maturation markers VGF, GAP-43, and tissue transglutaminase [48]. In turn, GDNF, a RET receptor agonist, was able to induce NB differentiation; still, the cotreatment results with ATRA were the same as any drug alone, suggesting that the RA and RET pathways share the downstream signalling elements [49].

While the inhibition of MAPK, PI3K, and RET pathways impairs RA-induced NB differentiation, inhibition of the protein kinase C (PKC) pathway has the opposite effect. Co-exposure of H7, PKC inhibitor, and ATRA resulted in increased neurite formation in non-MNA SH-SY5Y and MNA LA-N-5 NB lines [43]. Similarly, inhibition of tyrosine phosphatase resulted in potentiation of RA-induced differentiation of multiple NB lines. A combination of sodium orthovanadate and ATRA increased the length of the neurites by 15–90% and almost doubled the percentage of differentiating RA-sensitive SH-SY5Y, LAN-5, and KELLY cells [48]. Additionally, immunodetection revealed a significant increase in differentiation markers Trk, βIII-tubulin, pERK, pAKT, and p21, and a minor elevation of MYCN expression [48].

Another pathway, derivatives of which attenuate RA signalling, is arachidonic acid metabolic cascade. Lipoxygenase (LOX) pathway inhibition enhanced the pro-differentiation action of RA. Co-treatment of ATRA with caffeic acid or celecoxib—LOX and cyclooxygenase 2 (COX2) inhibitors—increased the expression of mature neuronal markers NF-200 and NeuN in MNA SK-N-BE(2) cells [50]. Interestingly, those cotreatments resulted in the decreased expression of NeuN marker in non-MNA SHSY5Y line. Meanwhile, the caffeic acid-RA combination resulted in a decrease in proliferation similar to RA treatment alone. Conversely, Celecoxib treatment led to the prominent cytotoxic effect in both cell lines, while the addition of ATRA increased the proliferation activity of SK-N-BE(2) but not SHSY5Y cells. 

The pathways mentioned previously are essential for RA’s effects or counteracting RA signalling, yet activation of other pathways could also enhance RA actions on NB cells. For example, the activation of transforming growth factor β (TGF-β) signalling pathway by kartogenin allowed RA-resistant MNA IMR32 cells to undergo differentiation [51]. Additionally, there is evidence of vasoactive intestinal peptide (VIP) pathway involvement in RA-initiated signalling cascade. VIP cotreatment with ATRA or 9-cis RA led to the decreased MYCN expression in RA-resistant IMR32 cells [52]. 

While multiple pathways mediate RA effects, RA treatment could also affect other signalling cascades. As such, 72 h pre-incubation with 9-cis RA predisposed non-MNA SH-EP, SH-SY5Y, and Kelly but not MNA LAN-5, SK-N-LO, and SK-N-FI NB cell lines to decreasing viability after subsequent exposure to STI-571, imatinib, a tyrosine kinase pathway inhibitor. This effect was not evident in cells which competed for differentiation or acquired RA resistance [53].

Finally, RA signalling might be modulated by non-specific compounds targeting proteasomes. The introduction of MG132, a protease inhibitor, resulted in increased apoptotic processes in RA-treated SK-N-BE(2) and SH-SY5Y cells; furthermore, surviving cells differentiated, obtaining the neuronal morphology, and stopped expression of stem markers PCNA, NF-κB, Sox2, Oct4, and Nestin [54].

**Table 2:** Summary of the drugs that activate cellular cascades.

Compound	Chemical Structure	Signalling Pathway	Target Protein	Model System	Markers	References
U0126	Aryl sulfide	MAPK inhibitor	MEK kinase	in vitro	ZNF423, TGM2, pERK, NRF2	[41,42]
PD98059	Monomethoxyflavone	MAPK inhibitor	MEK	in vitro	PML-NB formation	[43]
SB203580	Imidazole	MAPK inhibitor	Akt&p3	in vitro	NRF2	[41]
H7	Hydrochloride anileridine salt	MAPK inhibitor	PKC	in vitro	PML-NB formation	[43]
LY294002	Aryl sulfide	PI3K inhibitor	PI3K	in vitro	NRF2, p-AKT, p21, p27Kip	[41,45]
SP600125	Anthrapyrazole	JNK1/2 inhibitor	JNK1/2	in vitro	NRF2	[41]
Curcumin	Diferuloylmethane	JNK inhibitor	NFkappaB, AP-1, c-Jun N-term K	in vitro	GAP-43	[44]
CTNF	Peptide	Jak-STAT, MAPK, PI3K activator	CTNF receptor	in vitro	pSTAT3, pAkt, pERK1/2	[46]
STI-571, Imatinib	Benzamide 2-morpholinochromenone	Tyrosine kinase pathways inhibitor	abl, c-kit, PDGF-R	in vitro	-	[53]
Sodium orthovanadate	Vanadium oxoanion	Tyrosine phosphatase inhibitor	Tyrosine phosphatase	in vitro	Trk, βIII-tubulin pERK, pAKT, MYCN p21	[48]
ZD6474, Vandetanib	4-anilinoquinazoline	Growth factor pathways inhibitor	VWGFR2, EGFR, RET	in vitro	pRET	[47]
VIP	Peptide	VIP signalling pathway	VIP receptors	in vitro	MYCN	[52]
D4 aptamer	93-base 2′-fluoro-RNA-based aptamer	RET	RET receptor inhibitor	in vitro	VGF,GAP-43, tissue transglutaminase	[49]
GDNF	Protein	RET	RET receptor agonist	in vitro	-	[49]
Kartogenin	2-([1,1′-Biphenyl]-4-ylcarbamoyl)benzoic A	TGF-β signalling activator	Filamin A	in vitro	-	[51]
Caffeic acid	Hydroxycinnamic A derivative	Processing of arachidonic acid	5-LOX	in vitro	NF-200, NeuN	[50]
Celecoxib	NSAID with diaryl-substituted pyrazole ring	Processing of arachidonic acid	COX2	in vitro	NF-200, NeuN, CRABP-1	[50]
MG132	Synthetic peptide aldehydes	Protein degradation inhibitor	Proteasome	in vitro	phospho-histone H, PCNA, NF-κB, Sox2, Oct4, Nestin	[54]

### 3.7. Epigenetics and RA Signalling

Epigenetics refers to the reversible alterations to the gene expression in response to chemical changes of DNA without changing nucleotide sequencing and histones, the proteins binding DNA and supporting DNA coiled structure. The two most common types of drugs that work through epigenetic modulation are histone deacetylases (HDACs) and methylation inhibitors.

#### 3.7.1. HDAC Inhibitors 

HDACs affect gene expression by inhibiting histone deacetylases, which increases the acetylation of the histones. After accepting an acetyl group, histones typically bind DNA strand with lesser affinity. This, in turn, liberates DNA regions and allows for the initiation of the transcription. 

Changes in gene expression ultimately affect all cell processes, so drugs targeting epigenetic modifications might affect cellular response to RA treatment [55]. Thus, treating SH-SY5Y cells with ATRA and HDAC inhibitors trichostatin A, sodium butyrate, and suberoylanilide hydroxamic acid resulted in a robust dose-dependent decrease of cell viability and proliferation [56]. Besides, co-exposure to sodium butyrate and ATRA decreased the expression of cancer markers MYCC (3-fold) and Bmi1 (2-fold) while also downregulating the differentiation markers β-3 tubulin (2-fold) and NeuN (2-fold) in SH-SY5Y and SK-N-BE cell lines [57]. Selective HDAC1 inhibitors BRD8430 and compound 60 affected NB cell lines in the same way. Combined with 13-cis RA, those drugs increased the differentiation score of Kelly and BE(2)-C cells by 25% compared to a mono treatment [58]. 

In turn, compounds targeting the HDAC8 demonstrated a pro-differentiating action. Combined treatment with PCI-48012 and ATRA increased the neurite formation in BE(2)-C cells and IMR-32 by 150% and 50%, respectively. Additionally, the drug combination decreased the MYCN expression by 60% compared to RA alone in BE(2)-C cell line [59]. Furthermore, the drugs retained their anti-tumour effect in vivo. Administered to the BE(2)-C xenografted mice PCI-48012 combined with 13-cis RA resulted in a more prominent decrease in tumour volume than the single drug alone. A similar synergy in decreasing NB colony formation was observed between ATRA and TH34, a small-molecule HDAC inhibitor [60]. Another HDAC inhibitor CBHA was administered to SMS-KCN-69 xenografted mice demonstrated synergistic effects when co-treated with ATRA supporting the beneficial effects of HDAC inhibitors *in vivo*. The tumour burden was decreased with minor animal toxicity, the specificity of the response attributed to the increased histone acetylation [61].

#### 3.7.2. Methylation Inhibitors

Methylation of the histones results in the opposite effects that of acetylation. Methylated histones become bound to DNA more tightly, thereby restricting the binding of transcription machinery to the gene promotor sequences, silencing gene expression. The inhibition of histone methylation restores the transcription of the affected genes.

In addition to inhibiting histone deacetylase, activation of transcription could be achieved though inhibiting methylation. This is the mechanism of action of GSK-J4, an inhibitor of H3K27 and JMJD3 demethylases. Treatment by each of them contributed to a more prominent decrease in cellular viability than ATRA treatment alone [62].

Another demethylating agent, 5-Aza-deoxycytidine (AZA), enhanced RA actions both in vitro and in vivo [57]. Exposure of SH-SY5Y and SK-N-BE cells to this drug and ATRA resulted in a 200% decrease in cell population doubling and a 50% decrease in MYCC levels in both cell lines; interestingly, MYCN expression remained unaltered. The expression of the neuronal markers Bmi1, NeuN, and βIII-tubulin differed between the cell lines. While in SK-N-BE(2) cells all the three of the markers were downregulated by 50% compared to no treatment, the SHSY5Y cells displayed a similar decrease in b-tubulin, no change in Bmi1, and a 40% increase in NeuN levels [57]. When testing this drug in mice xenografted with SK-N-AS or LAN5 lines, AZA + ATRA administration led to decreased tumour growth and increased animal survival [63].

### 3.8. Other Drugs That Exhibit Synergy with RA

This section of the review focuses on the interactions between RA and drugs used in clinical practice, examining the combinations showing the most potent differentiating or pro-apoptotic actions. For example, the co-treatment of ATRA and herbimycin A resulted in a marked increase in NB differentiation in vitro [15]. Contrarily, an immunostimulant polyinosinic-polycytidylic acid enhanced anti-proliferative and pro-apoptotic actions of 13-cis RA, decreasing cell viability by 30% and increasing the number of apoptotic cells by 200% compared to 13-cis RA treatment alone [64]. The expressions of apoptotic markers (TLR3, pIRF3, and MAVS—10% increase; apoptotic protein cleaved-PARP—50% increase) were increased in treated SK-N-DZ cells. Additionally, when administered to SK-N-DZ—xenografted mice—concurrently, this drug slowed down the tumour growth.

Substantial evidence supports the synergy between RA and IFNγ. The initial studies showed that co-treatment of IFN-γ with ATRA led to a greater decrease of *MYCN* mRNA in LAN5 cells compared to either treatment alone [65]. Subsequent studies confirmed the IFN-γ–ATRA synergistic inhibition of MYCN in LAN5 cells, accompanied by a 1.5-fold increase in differentiated cell number and a 20-fold increase in neurite outgrowth index [66]. The IFN-γ–ATRA combination induced enhanced differentiation of IMR32 and LAN 2 cells and increased the neurite outgrowth in SH-SY-5Y, IMR-32, LAN1, and LAN2 cells. Surprisingly, those marked changes were not accompanied by a decrease in MYCN expression in IMR-32 cells. All the exposed cell lines displayed growth inhibition to some degree, yet compared to other tested cell lines, the SH-SY-5Y cell line demonstrated the least benefit from the combinational treatment [67]. The observed efficiency of IFN-γ + RA on MNA cell lines was associated with enhanced degradation of MYCN protein [66].

Similarly, a synergy between RA and dehydroepiandrosterone (DHEA), a steroid hormone precursor was reported. Co-administration of those drugs resulted in decreased cellular movements, more prominent neurite elongation, more significant cell proliferation arrest, and increased expression of GAP-43 in SK-N-BE cells [68]. Finally, the study testing the clinically relevant cytotoxic drugs revealed the 0.01 mmol vincristine–10 mmol 13-cis RA combination to be the most effective at inducing cell apoptosis and inhibiting MYCN expression in Kelly cells [69]. While some differentiation was observed, the number of differentiated cells was lower than in 13-cis RA treatment alone. The same study showed another combination of note—cisplatin–RA (0.5 μmol—10 mmol). This drug combination induced a strong cytotoxic effect, inducing both necrosis and apoptosis but retaining some expression of MYCN.

### 3.9. NB Research Models for RA Studies

The majority of the reviewed studies investigated RA effects in vitro by utilising traditional 2D NB cell models. Table 3 summarised the impact of RA and investigated treatments on cell morphology. SH-SY-5Y was the most commonly used NB cell line. Derived from SK-N-SH cells, this cell line readily obtains neuronal-like phenotype as a result of RA exposure. SH-SY-5Y cells carry only a single copy of MYCN. This oncogene promotes proliferation and cell cycle progression and dedifferentiation into stem cell-like state. The functions of MYCN explains why its amplification, often present in NB tumours, is associated with poorer patient prognosis [70]. Several MYCN amplified NB cell lines were used in the studies including Kelly, LAN1, LAN3, SK-N-BE (2), and IMR-32. In addition to variation in MYCN expression, NB cell lines also differ in the expression of other cellular proteins, including p53 and tyrosine hydroxylase and their sensitivity to the cytotoxic drugs. Of the latter, CHLA cell lines are a prominent example of the resistance to the multiple cytotoxic drugs commonly used in NB therapy [71].

Traditional 2D cell cultures, while being an affordable mean of screening for the potential compounds of interest, fail to reproduce the complexity of the NB at the tissue level. One of the main reasons for this model inadequacy is limited interactions between cultured cells and a complete absence of the interactions with other cell types and extracellular matrix. Those interactions were found to be extremely important in NB differentiation and progression [72,73]. The more complex in vitro system that could account of those deficiencies would be a 3D models, which could be populated both by NB cells, supporting cells, and cells of the immune system [74]. Systems like that would have been able to reproduce the tumorigenic environment. Unfortunately, this is still the developing field and, despite its promise, the studies focusing on RA signalling have not adopted that model yet.

**Table 3:** In Vitro models and compound testing.

Cell line	Treatment/Drug (s)	Dosage	Phenotype	ATRA Sensitive (Y/N)	MYCN Status	Reference
SH-SY5Y	9-cis RA	10 μM	Neurite form.	Y	Non-amp	[20]
	LG153	1 μM	↑ neurit. growth			[20]
	9-cis-UAB30	10 µM	↑ neurit. growth			[30]
	R116010	1 μm	-			[38]
	Minocycline	100 μm	-			[39]
	Ro 13-6307	1 μM	Neurite form			[24]
	GGA	10 μM	Neurite form			[33]
	Fenretinide	3 μM	-			[28,29]
	Calcitriol	10 nM	-			[40]
	24,25(OH)2D3	10 nM	-			[40]
	KH 1060	10 nM	-			[40]
	EB 1089	10 nM	-			[40]
	Trolox	200 μM	-			[41]
	n-acetyl-cysteine	5 mM	-			[41]
	BSO	0.5 mM	-			[41]
	UO126	10 μM	No diff.			[41]
	SB203580		-			[41]
	SP600125		-			[41]
	LY294002	20 μM	No diff.			[41]
	PD98059	25 μM	No diff.			[43]
	H7	25 μM	↑ diff.			[43]
	Curcumin	10 μM	↑ neurit. growth			[44]
	CNTF	5/50/500 nM	↑ neurit. growth			[46]
	ZD6474	5 μM	↑ neurit. growth			[47]
	Sodium orthovanadate	5 μM	↑ diff. and neurit. growth			[48]
	Caffeic acid	13/52 μM	Differentiation			[50]
	Celecoxib	10/50 μM	Differentiation			[50]
	STI-571	1/20 μM	-			[53]
	MG132	500 nM	Apoptosis and diff			[54]
	Trichostatin A	200/500 nM	↓ neurit. length			[56]
	Sodium butyrate	2/5 mM	↓ neurit. length			[56]
	Suberoylanilide hydroxamic acid	1/2 μM	↓ neurit. length			[56]
	5-Aza	1 μM	-			[57]
	IFN-γ	100 U/mL	neurit. length			[67]
shNF1 SH-SY5Y	U0126	1/2.5/5 μM	-	N	Non-amp	[42]
SK-N-SH	LY294002		No diff.	Y	Non-amp	[45]
	Herbimycin A	236 nM	Diff.			[15]
SK-N-AS	9-cis-UAB30	10 µM	↑ neurit. growth	N	Non-amp	[30]
	6-Methyl-UAB30	25 μM	↑ neurit. growth			[31]
	Sodium orthovanadate	5 μM	↑ diff. and neurit. growth			[48]
	GSK-J4	1 μM	-			[62]
	5-Aza	2 μM	Neurite form.			[63]
SH-EP	9-cis-UAB30	10 µM	↑ neurit. growth	N	Non-amp	[30]
	6-Methyl-UAB30	25 μM	↑ neurit. growth			[31]
	R116010	1 μm	-			[38]
	STI-571	1/20 μM	-			[53]
SK-N-BE(2)	4-oxo-13-cis RA	10 μM	Neurite form.	Y	Amp	[23]
	Ro 13-6307	1 μM	Neurite form.			[24]
	Fenretinide	3 μM	-			[28,29]
	9-cis-UAB30	10 µM	↑ neurit. growth			[30]
	6-Methyl-UAB30	25 μM	↑ neurit. growth			[31]
	LY294002		No diff.			[45]
	ZD6474	5 μM	↓ neurit. growth			[47]
	D4 aptamer		↓ neurit. growth			[49]
	GDNF	100 ng/mL	Neurites form.			[49]
	Caffeic acid	13/52 μM	Diff.			[50]
	Celecoxib	10/50 μM	Some diff.			[50]
	MG132	500 nM	Apoptosis and diff			[54]
	Sodium butyrate	1 mM	-			[57]
	5-Aza	1 μM	-			[57]
	BRD8430	0.6 μM	↑ diff.			[58]
	compound 60	0.3 μM	↑ diff.			[58]
	PCI-48012	30 μM	-			[59]
	TH34	5 μM	↑ neurit. form.			[60]
	GSK-J4	1 μM	-			[62]
	DHEA	100 μM	↑ neurit. form.			[68]
Kelly	Sodium orthovanadate	5 μM	↑ diff. and neurit. length	Y	Amp	[48]
	STI-571	1/20 μM	-			[53]
	BRD8430	0.6 μM	↑ diff.			[58]
	compound 60	0.3 μM	↑ diff.			[58]
	Herbimycin A	236 nM	Diff.			[15]
	Vincristine	0.01 mM	Apoptosis			[69]
LAN1	5-Aza	2 μM	Neurite form.	Y	Amp	[63]
	IFN-γ	100 U/mL	Diff.			[15]
LAN2	IFN-γ	100 U/mL	No diff.	Y	Amp	[15]
LAN5	9-cis RA, ATRA	20 μM	Neurite form.	Y	Amp	[18]
	All-trans-retinoyl β-glucuronide	10 μM	Neurite form.			[34]
	Fenretinide	3 μM	-			[27,28]
	PD98059	25 μM	No diff.			[43]
	H7	25 μM	↑ diff.			[43]
	Sodium orthovanadate	5 μM	↑ diff. and neurite length			[48]
	STI-571	1/20 μM	-			[53]
	IFN-γ	100 U/mL	↑ diff.			[65,66,67]
LAN6	R116010	1 μm	-	Y	Non-Amp	[38]
IMR32	R116010	1 μm	-	Y	Amp	[38]
	Fenretinide	3 μM	-			[29]
	9-cis-UAB30	10 μM	↑ neurit. growth			[30]
	Kartogenin	1/5/10 μM	Apoptotic shape			[51]
	Vasoactive intestinal peptide	1 μm	Diff.			[52]
	PCI-48012	30 μM	↑ diff.			[59]
	IFN-γ	100 U/mL	↑ diff.			[65,66]
NGP	R116010	1 μm	-	Y	Amp	[38]
	Fenretinide	3 μM	-			[29]
SMS-LHN	4-oxo-13-cRA	10 μM	Neurite form.	Y	Non-AMP	[23]
	Fenretinide	3 μM	-			[27]
SMS-KCN	4-oxo-13-cRA	10 μM	Neurite form.	Y	Amp	[23]
SMS-KCNR	4-oxo-13-cRA	10 μM	Neurite form.	Y	Amp	[23]
	Fenretinide	3 μM	-			[27]
CHLA-20	4-oxo-13-cRA	10 μM	Neurite form.	N	Non-Amp	[23]
CHLA-79	4-oxo-13-cRA	10 μM	Neurite form.	Y	Non-Amp	[23]
WAC(2)	9-cis-UAB30	10 µM	↑ neurit. growth	Y	Amp	[30]
	6-Methyl-UAB30	25 μM	↑ neurit. growth			[31]
CHLA-90	GSK-J4	1 μM	-	Y	Non-Amp	[62]
CHP-212	5-Aza	2 μM	Neurite form.	Y	Amp	[63]
GIMEN	R116010	1 μm	-	Y	Non-Amp	[38]
Neuro2a ^a^	Retinoic Acid-Triazolyl Derivatives	10/20 μm	Neurite form. and apoptosis	Y	*	[32]
MCF-7 ^b^	4-HBT(TNPB)	10 nM	-	Y	NA	[37]

↑ Increased. ↓ Decreased. ^a^ murine NB cell model. ^b^ breast adenocarcinoma cell model. * Expresses the human *MYCN* transgene [75].

After determining the compound’s action in vitro, the subsequent step is to verify its efficiency in vivo. The reviewed studies utilised the murine xenograft models using either athymic (nu/nu) or severe immunodeficiency strain, allowing them to receive the transplanted NB cells, usually SH-SY-5Y, LAN5, and BE(2)-C cell lines (Table 4). After administration, NB cells were able to proliferate and form tumour masses. This model system allows analysing the interactions between compounds of interest, NB cells, and other tissues, partially reproducing the human patients’ physiologic conditions. Another benefit of the murine models is determining the drug’s pharmacokinetic profile, as the most efficient drug in vitro might be metabolised too rapidly or produce toxic derivatives to have any clinical potential. The limitation of the murine studies is the difference in the drug metabolism; drugs successful in animals have been found ineffective in humans and drugs exhibiting toxic effects on mice have more potential in human patients [76].

### 3.10. RA Drug Delivery Systems

RA has limited clinical efficiency and is suspect to drug resistance due to hydrophobic nature, short half-life, varying plasma concentration between patients, and cytotoxicity. To overcome those limitations, two approaches are being investigated, using RA compounds in combination treatments (Table 1) and improving RA drug delivery systems (DDS). The use of alternative DDS for differentiation therapy has recently been reviewed in other cancers, such as acute promyelocytic leukaemia (APL) [77]. Currently, oral administration of RA is the first-choice treatment in neuroblastoma patients. Two studies have been completed assessing alternative RA drug delivery systems in NB. The first was a pre-clinical evaluation of high density lipoprotein (rHDL) nanoparticles which encapsulated fenretinide (FR) for the treatment of neuroblastoma [78]. When comparing the cytotoxicity of free FR against rHDL/FR using NB cell lines SK-N-SH and SMS-KCNR, there were 2.8 and 2-fold lower IC50 values for the incapsulated FR. The IC50 value of free FR was <40 times that of rHDL/FR in the non-malignant control retinal pigment epithelial cells (ARPE-19). This data experimentally verified the selective anti-cancer impact of rHDL on malignant cells. The observed selectivity is due to FR uptake from rHDL nanoparticles by the SR-B1 receptor that is likely to be overexpressed in NB. This study concluded the in vitro selective therapeutic efficiency of the rHDL nanoparticles was 100-fold upon encapsulation of the drug.

A more recent study investigated the efficiency of a multifunctional nanobiohybrid material composed of Ag@Bi2Se3/RNA [79]. This nanoparticle is a fluorescent tagged three-way Junction/miRNA-17/RA to induce neuroblastoma differentiation, by the slow release of RA into the cytosol and the inhibition of miR-17 using a displacement-of-function strategy. After six days of treatment SH-SY5Y cancer cells fully differentiated into neuron- like cells and ceased growth without requiring sequential interventions. This efficiency has not previously been reported for neuroblastoma differentiation therapy. However, no alternative delivery systems are being tested at clinical trial for neuroblastoma treatment.

## 4. Clinical Trials on RA

RA isomers’ success as pro-differentiation agents in preclinical research opened opportunities to advance them to NB clinical trials (Table 5). ATRA and 9-cis RA isomers can induce NB cell differentiation at smaller concentrations than 13-cis RA. However, compared to ATRA and 9-cis RA, 13-cis RA, or isotretinoin, exhibited more favourable pharmacokinetic properties. In a phase I trial, 13-cis RA reached a higher peak concentration while having a much longer half-life than other isomers tested; additionally, its levels remained consistent during the treatment [80]. CCG-3891 clinical study was the most extensive study investigating the efficiency of 13-cis RA in NB treatment. While the main focus of this trial was comparing the efficiency of myeloablative therapy supported by autologous bone marrow transplantation and non-myeloablative chemotherapy, the study’s additional goal was to determine the efficiency of isotretinoin in eradicating the minimal residual disease. Therefore, after completing the primary therapies, 258 of the 539 initial patients were randomized into two groups. The experimental group members were assigned six cycles of 13-cis-retinoic acid (160 mg/m^2^/day, administered orally in two divided doses) for 14 consecutive days in a 28-day cycle. The participants in the control group received no supplemental therapy. The patients who received 13-cis RA treatment had a significant increase in 3-year event-free survival rate (46 ± 6 per cent compared to 29 ± 5 per cent).

Furthermore, this effect was observed in both transplantation and chemotherapy cohorts [81]. A subsequent retrospective cohort study, focusing on the long-term effects of the treatment, supported the benefit of 13-cis supplementation due to the observed increase in the patients’ 5 years survival rate in the treated group [82]. However, the overall survival difference was found to be non-significant in both studies [81,82]. The observed benefit of isotretinoin supplement was registered only in the group with minimal residual disease. In patients in partial remission after initial chemotherapy, the 13-cis treatment was ineffective [81].

The efficacy of 13-cis RA treatment alone in treating NB might be even more limited, as supported by the subsequent study focused on 175 high-risk stage 4 NB patients. The obtained data displayed no significant difference in 3-year event-free survival rates between patients receiving the RA therapy and patients treated with placebo, yet those results could be attributed to the suboptimal amount of the administered compound [83]. Nevertheless, isotretinoin is the only RA form in current clinical practice and prescribed for 6 months of post-consolidation therapy.

While most RA clinical trials focused on the 13-cis RA isoform, a phase II clinical trial investigated the efficacy of ATRA-IFNα2a combination. Sixteen NB patients were administered ATRA (orally 90 mg/m^2^/day in three divided doses, three consecutive days per week) and IFNα2a (subcutaneously, 3 × 10^6^ U/m^2^/day, five consecutive days per week) in four cycles). The study determined complete response as normalization of urinary catecholamines, complete resolution of all soft tissue tumours, and partial response as a 50% reduction of all measurable tumour. Unfortunately, the participants failed to benefit from the treatment, suggesting the lack of ATRA-IFNa2 efficacy in NB [84].

The combination of differentiating and antibody-based therapies may work together well tackling tumour through different pathways. Here, we will discuss those studies that assessed efficacy of anti-GD2 antibody in a combination with isotretinoin. Several therapeutic antibodies directed against disialoganglioside GD2, an antigen highly expressed on neuroblastoma cells have been developed, tested in clinical trials, and reviewed [85]. A phase III study examined the effect of isotretinoin and the human/mouse chimeric antibody, ch14.18 (also known as dinutuximab), in conjuncture with granulocyte-macrophage colony-stimulating factor (GM-CSF) and IL-2 on the improving NB outcomes [86]. The study included 226 participants, 113 of whom were assigned to the standard treatment group consisting of a 160 mg/m^2^/day of isotretinoin for 14 days in six 4-week cycles (Table 5, NCT00026312). One-hundred and thirteen patients in the immunotherapeutic group received 25 mg/m^2^/day of ch14.18 for 4 days during five 4-week cycles; they were additionally supplemented with isotretinoin (160 mg/m^2^/day for last 2 weeks of each cycle) and Il-2 (3.0 × 10^6^ IU/m^2^/day for 4 days in week 1 and 4.5 × 10^6^ IU/m^2^/day for 4 days in week 2). This treatment was beneficial for the patients in immunotherapy group, who experienced increased rates of event-free survival (66 ± 5% vs. 46 ± 5%) and overall survival (86 ± 4% vs. 75 ± 5%) at 2 years compared to the patients in standardised treatment group. Despite the effectiveness of the treatment, 52% of the patients experienced significant neuropathic pain of grade 3, 4, and 5 [87]. This discovered toxicity necessitated the development of a new treatment schedule, which consisted of a continuous ch14/18 long-term infusion over 10 days (10 × 10 mg/m^2^/day) and isotretinoin (160 mg/m^2^/day for 2 weeks) and IL-(6.0 × 10^6^ IU/m^2^/day for days 1–5 and 8–12) for 5 or 6 cycles [88]. A subsequent phase II clinical trial compared 53 patients following the long-term infusion protocol to the matched historical control group from AIEOP database (Italian Pediatric Ematology and Oncology Association) [89]. The patients’ response rate was 40.5%; the 4-year progression free survival was 33.1%; 4-year overall survival was 47.7%. The effectiveness of the treatment coupled with the significantly decreased pain scores and adverse effect profiles signifies the strong potential of long-term infusion of the ch14.18–isotretinoin–IL2 combination in NB treatment.

Another anti-GD2 antibody of interest is a murine 3F8 antibody. Its properties were determined in Phase III trial, which included 169 children with refractory stage 4 NB in first remission [90]. The patients were divided into three cohorts (Table 5, NCT0118389). The first received only 3F8 (10 mg/m^2^), the second—3F8 + IV GM-CSF + 13 cis-RA RA (10 mg/m^2^, 250–500 ug/m^2^, 160 mg/m^2^), and the third—3F8 + SC GM-CSF + 13-cis RA (20 mg/m^2^, 250–500 ug/m^2^, 160 mg/m^2^). The latter group also had 28 patients deemed ultra-high-risk, who received additional induction therapy. The patients in the third cohort received the most benefit from the treatment, as their 5-year progression free survival was 62%, while the overall survival reached 81% (compared to 44%/49% and 56%/61% of cohorts 1 and 2), the results demonstrating that anti-GD2 3F8 antibody–GM-CSF–13-cis RA polytherapy is effective against minimal disease.

Fenretinide, a derivative of RA, exhibited a potent cytotoxic effect on NB cells, as discussed in Section 3.3. Following the promising results of preclinical studies and a phase I trial, fenretinide’s efficiency in treating patients with refractory/resistant high-risk neuroblastoma was tested in a phase II trial [91]. Sixty-two patients received oral capsules; those ≤18 years of age received a dose of 2475 mg/m^2^/day divided into three equal doses, while those >18 years of age were treated with 1800 mg/m^2^/day divided into two equal doses for seven days followed by two weeks rest. For statistical analysis, the patients were stratified based on their initial NB presentation. Stratum I was composed of 38 patients with a CT/MRI measurable tumour; 24 patients without a CT/MRI measurable tumour but exhibiting a MIBG avid tumour and/or a tumour in the bone marrow were assigned to stratum II. Fenretinide treatment resulted in one partial response in stratum II and 13 cases of a prolonged stable disease divided between strata (seven patients in stratum I and six patients in Stratum II), affecting 24% of exposed patients. Hence, this compound failed to meet the protocol criteria for efficacy, attributing to its poor bioavailability [91]. An attempt to design a more effective fenretinide formulation resulted in creating the oral drug/powdered lipid complex (fenretinide/LXS). This complex was found to cause higher plasma levels than the original oral capsules while exhibiting minimum toxicity [92]. Its efficiency is still to be determined in subsequent trials. Besides testing this formulation alone, phase I studies were conducted to assess the potential of fenretinide/LXS for multidrug therapy and further address its pharmacokinetic profile and toxicity. This clinical trial explored the potential synergy between fenretinide/LXS, ketoconazole, a drug inhibiting fenretinide metabolism, and cytotoxic drug vincristine (Table 5, NCT02163356). The results of this study are not available yet.

There is a lack of clinical trials focusing on the potential combinations of RA and synergistic drugs. A phase I clinical trial investigating the combination of RA and ZD6474, an inhibitor of VEGF signalling, was commenced in 2007 but was terminated due to the lack of enrolment (Table 5, NCT00533169). Clinical trials determining interactions between RA and histone deacetylase inhibitor vorinostat were more successful. Two-phase I clinical trials, aiming to determine the effective concentration of the drugs, have been initiated; their results have not been reported yet (Table 5, NCT01208454, NCT00217412).

Despite the limited interest in investigating RA and synergistic drug combinations’ efficiency, the discovery of the novel potent anti-cancer meditation might give rise to the future RA clinical trials. Many in vitro studies confirmed the synergic effect of interaction between the RA signalling and all mentioned pathways. Those drugs might be more efficient in combination with RA and give rise to the more potent NB treatment. Currently, several phase I clinical trials recruit NB patients to investigate the compounds affecting MAPK, PI3K, ERK1/2, and proteasomal signalling.

**Table 5:** Summary of Pivotal Clinical Trials for RA use in Neuroblastoma.

Trial Number	Study Title	Treatment	N	Trial Phase	Outcomes	Status	Results Published
CCG-3891	Conventional Dose Chemoradiotherapy vs. Ablative Chemoradiotherapy With Autologous BMT for High Risk Neuroblastoma	13-cis RA	539	III	Event-free survival rate, overall survival rate	Complete	[81,82]
ENSG clinical trial	European Neuroblastoma Study Group clinical trial	13-cis RA	175	III	Event-free survival rate	Complete	[83]
CCG-0961	Phase 2 All Trans Retinoic Acid/Interferon alpha 2A	ATRA + IFNα2a	16	II	Normalization of urinary catecholamines, significant reduction of tumour size	Complete	[84]
NCT00053326	Fenretinide in Treating Children With Recurrent or Resistant Neuroblastoma	Fenretinide	59	II	Progression-free survival, overall survival	Complete	[91]
NCT00295919	N2004-04: Fenretinide LXS in Treating Patients With Recurrent, Refractory, or Persistent Neuroblastoma	Fenretinide/LXS +/− Ketoconazole	32	I	Peak plasma concentrations, maximum tolerated dose	Complete	[92]
NCT02163356	Fenretinide Lym-X-Sorb + Ketoconazole + Vincristine for Recurrent or Resistant Neuroblastoma (SPOC2013-001)	Fenretinide/LXS + Ketoconazole + Vincristine	4	I	Peak plasma concentrations, maximum tolerated dose	Complete	NP
NCT00026312	Isotretinoin With or Without Dinutuximab, Aldesleukin, and Sargramostim Following Stem Cell Transplant in Treating Patients With Neuroblastoma	Dinutuximab/GM-CSF/IL-2/13-cis RA	226	III	Event-free survival rate, overall survival rate	Complete	[87]
NCT02743429	Phase II Study of Monoclonal Antibody ch14.18/CHO Continuous Infusion in Patients With Primary Refractory or Relapsed Neuroblastoma	Dinutuximab/IL-2/13-cis RA	53	II	Progression free survival, overall survival	Complete	[89]
NCT01183897	3F8/GM-CSF Immunotherapy Plus 13-Cis-Retinoic Acid for Primary Refractory Neuroblastoma in Bone Marrow	anti-GD2 3F8 antibody/GM-CSF/13-cis RA	169	III	Progression free survival, overall survival	Complete	[90]
NCT00533169	ZD6474 Alone and in Combination With Retinoic Acid in Pediatric Neuroblastoma	ZD6474 + 13-cis RA	10	I	Peak plasma concentrations, maximum tolerated dose	Terminated	NP
NCT01208454	Vorinostat and Isotretinoin in Treating Patients With High-Risk Refractory or Recurrent Neuroblastoma	Vorinostat + 13-cis RA	29	I	Peak plasma concentrations, maximum tolerated dose	Complete	NP
NCT00217412	Vorinostat With or Without Isotretinoin in Treating Young Patients With Recurrent or Refractory Solid Tumors, Lymphoma, or Leukaemia	Vorinostat + 13-cis RA	60	I	Peak plasma concentrations, maximum tolerated dose	Complete	NP

NP data not published.

## 5. Conclusions

Neuroblastoma is a highly heterogeneous solid malignancy that consists of neuroblasts and undifferentiated Schwann cell precursors. This offers a window of opportunities to develop a pro-differentiating and anti-proliferative therapy for high-risk NB; however, a systems biology approach to devising such strategies is currently missing.

We attempted to systematically review current efforts to differentiate neuroblastoma directly targeting the classical retinoic acid pathway and indirectly by mimicking their downstream effects of RA published from 1 January 1980 to 1 July 2020. The complexity of neuroblastoma biology makes it difficult to identify intercellular signals that could be either disrupted or induced, to drive tumour cells towards differentiation [72,93,94]. Similarly, signalling interactions between different components of the tumour microenvironment and their interactions with the tumour cells themselves remain poorly characterized, despite numerous focused efforts [95,96].

Currently, the only form of RA employed in clinical practice is 13-cis RA, also known as isotretinoin, as part of the treatment regimen for high-risk neuroblastoma patients; however, some patients display a varied response to the drug, with many being non-responsive to this therapy [10,12,17,97]. Furthermore, the use of this drug is limited to maintenance therapy, as there are no data on its efficacy in treating sustained tumours. Interestingly, 13-cis RA is the isoform with the lowest activity on the NB cells in vitro. The other two isoforms of RA, ATRA and 9-cis RA, exhibited much higher pro-differentiating and pro-apoptotic responses respectively. However, the subsequent in vivo studies established their unfavourable pharmacokinetic and toxic profiles, limiting their use as NB treatments. Despite the positive effects of differentiation therapy giving better survival scores than checkpoint inhibition, there are some side effects and tumour relapses which warrant the need for more physiological safe and persistent RA derivatives [98,99].

The only other derivative of RA to reach clinical trial is a cytotoxic compound fenretinide. Initially deemed inefficient due to low bioavailability, subsequent studies led to the development of a novel fenretinide/LXS formulation which achieved a higher drug plasma concentration. This compound is currently undergoing clinical trials which will evaluate its efficacy in NB treatment. This highlights the importance of further screening on retinoid chemical compounds with the aim of identifying less toxic and more potent pro-differentiating effects. To advance RA derivatives being identified and brought to clinical trial, there is a clear need for more effective pharmacokinetic mortification, to reduce drug toxicity and improve bioavailability. The success in vitro of alternative delivery systems at solving these limitations, such as the use of nanoparticles to administer RA therapy to neuroblastoma cell lines, demonstrates the potential of this area to increase the clinical application of RA therapy. Further investigation is required to access the use of established DDS in neuroblastoma differentiation treatment.

The most promising direction of RA therapy is the creation of synergistic multidrug treatments, composed of drugs that target different signalling pathways. This strategy aims to reduce drug resistance to standard care caused by the persistence of immature nerve cell precursors. The data suggest the synergistic interactions between RA and drugs in the following categories: MAP/PI3K/TGF-β agonists and CYP26/PKC/tyrosine kinase/proteosome inhibitors. Therefore, combining RA and several drugs from those classes might further promote their pro-differentiating actions in NB. Recent interest in the area of immunotherapy has led to the use of RA therapy in combination with anti-GD2 antibodies and IL-2, which has seen an improvement in neuroblastoma patient overall survival. In addition, there is strong evidence of the synergy between RA and epigenetic modulators, predominantly HDAC inhibitors, which were shown to induce NB differentiation in vitro and *in vivo*. To date nine clinical trials have taken place to determine the potential of this combinational treatment in high-risk NB patients. Further efforts are still required for more clinical validation to find better therapeutic regimens for high-risk NB patients.

Unfortunately, while the simultaneous usage of RA and other compounds to treat NB cells has been examined in vitro, there is a relative lack of further studies examining their interactions in vivo. As such, signalling cascade effector drugs have not been tested in combination RA in animal models or in 3D models, which could capture the NB tumour heterogenicity better than traditional 2D cell cultures [74]. Hence, the validation of the previous theoretical findings in models that are closer representations of the living organism is a next essential step to advance our understanding of neuroblastoma biology and to find better therapeutic combinations for high-risk NB patients.

To improve the survival score of RA as a monotherapy or in combination with targeted treatments, there is a need for a more personalised medicine approach—enabling patients to receive earlier diagnoses and optimal treatment regimes, ultimately leading to a change in clinical treatment practice from a trial-and-error approach to the right drug, for the right patient, at the right time [100]. To achieve this, more emphasis most be placed on profiling studies to decipher biomarkers for sensitivity or resistance to RA therapy in neuroblastoma patients. Recent studies identified possible predictive biomarkers of clinical response to retinoid differentiation therapy, enhancing the possibility of personalised RA therapy [101,102]. Further studies into differentiation therapy, drug combinations, and predictive biomarkers are required to tailor differentiating treatments to the individual characteristics of each patient, which should ultimately identify patients with more favourable outcomes to RA therapy.

## Figures and Tables

**Table 4:** Murine models used for RA studies.

Treatment (s)	Mouse Strain	Site of Injection	Cell Lines	Dosage	Efficacy	Reference
9-cis RA	HsdHan: RNU-rnu rats	Ectopic subcutaneous	SH-SY5Y	2 × 2.5 mg/day orally	↓ tumour V	[21]
9-cis RA	Rowett rnu/rnu mice	Ectopic subcutaneous	SH-SY5Y	5 mg/day orally	↓ tumour V&W, animal weight loss	[22]
Ro 13-6307	Rowett rnu/rnu mice	Ectopic subcutaneous	SH-SY5Y	0.3 mg/day orally	↓ tumour V&W, ↓ animal weight	[22]
Ro 13-6307	HsdHan: RNU-rnu rats	Ectopic subcutaneous	SH-SY5Y	0.12 mg/day orally	↓ tumour V&W, ↑ animal weight gain	[24]
R116010. RA	CD-1 nu/nu mice	Ectopic subcutaneous	SH-SY5Y	1.25/2.5 mg/kg 100 mg/kg	↑ RA concentration	[38]
All-trans-retinoyl β-glucuronide	CD-1 nu/nu mice	Ectopic subcutaneous	LAN5	25 μmol/kg; 30 μmol/kg	↓ tumour incidence and growth rate	[34,35]
PCI-48012 13-cis RA	NMRI nude mice	Ectopic subcutaneous	BE(2)-C	40 mg/kg/day 10 mg/kg/day	↓ tumour growth rate	[59]
CBHA ATRA	SCID mice	Ectopic subcutaneous	SMS-KCN-69	50/100/200 mg/kg 2.5 mg/kg	↓ tumour growth rate	[62]
5-Aza RA	Crl:Nu (Ncr)-Foxn1 nu mice	Ectopic subcutaneous	SK-N-AS, LAN-1	0.1 mg/kg/day 10 mg/kg/day	↓ tumour growth rate, ↑ survival	[63]
PIC 13-cis RA	NOD/SCID mice	Ectopic subcutaneous	SK-N-DZ	10 mg/kg 5 mg/kg	↓ tumour growth rate	[64]

↑ Increased. ↓ Decreased. V—volume. W—weight.

## Data Availability

Data is contained within this article.
